# Twelve Consecutive Laparotomies

**Published:** 1890-08

**Authors:** Virgil O. Hardon

**Affiliations:** Professor of Obstetrics and Diseases of Women and Children, Atlanta Medical College, Atlanta, Ga.


VOL. VII. AUGUST, 1890. NO. 6.

### Original Communications.

### TWELVE CONSECUTIVE LAPAROTOMIES.

By VIRGIL O. HARDON, M. D.,
Professor of Obstetrics and Diseases of Women and Children, Atlanta Medical College, Atlanta, Ga.

CASE XI.*—EXPLORATORY INCISION. RECOVERY.—M. P., married, age 28, nullipara. First noticed a tumor in the right lumbar region about two years ago, which gradually increased until it extended into the umbilical and right inguinal regions, and filled up the whole right side of the abdomen. No pain and only slight tenderness. General health has recently begun to fail. Examination showed the tumor to be solid, apparently connected with the liver, and only slightly movable. Could not be felt from the vagina. No diagnosis made.

*Operation October* 3, 1889.—Present, Drs. Westmoreland, Harris and Johnson. Incision in median line. Exploration showed the tumor to be an enormously enlarged liver. No operation

*Numbering continued from previous series.

attempted. Abdomen closed without washing or drainage. Recovery uneventful. Highest temperature 100°, on the evening of the second day. In January, 1890, patient reported that the tumor was in the same condition, but that her general health had much improved, which she attributed to the exploratory incision. It was probably mere coincidence.

CASE XII —BATTEY'S OPERATION. RECOVERY.—N. M., unmarried, age 28. For five years has suffered with severe pain in the ovarian regions, increased at the menstrual periods. This has become so severe and has so impaired her general health, that for the last three years she has been bedridden. Excessive hyperæsthesia of the whole abdomen is present. Examination under ether revealed no cause for her condition. All other modes of treatment having failed, Battey's operation was determined upon as a last resort.

*Operation December* 18, 1889.—Present, Drs. Harris, McRae and Murphey. Incision in the median line. Both ovaries removed. No adhesions, no appearance of disease in the organs. Abdomen closed without washing or drainage. Time of operation, 35 minutes. Recovery uninterrupted. Highest temperature 100.4°, on the evening of the second day. Patient returned home in four weeks completely relieved of her abdominal pain, and there has been no return of it up to the present time (June, 1890). She is completely restored to health in all respects.

CASE XIII.—BATTEY'S OPERATION. RECOVERY.—L. B., married, age 38, nullipara. For several years has suffered with pelvic pain, most severe at menstrual periods. Walking and sitting increase the pain. Menses irregular. Her condition has incapacitated her for her work, on which she is dependent for her living. Examination showed an enlarged and very tender ovary, prolapsed into Douglas' pouch and immovably fixed in that position.

*Operation December* 17, 1889.—Present, Drs. Harris and Elkin, and the students of the Atlanta Medical College. Incision in median line. Both ovaries removed. Abdomen closed without washing or drainage. Time of operation, 25 minutes. Patient made a good recovery with the exception of a slight stitch abscess. She was completely relieved of her former pain, and is now able to earn her living. She has, however, a small ventral hernia at the site of the stitch abscess which necessitates her wearing a pad and bandage.

CASE XIV.—SARCOMA OF RIGHT OVARY. OVARIOTOMY. RECOVERY.—P. W., age 54, widow, multipara. Menopause occurred six years ago. Has always had good health until April, 1889, when she noticed a lump in the right side, which gradually increased in size. About two months later she noticed a general enlargement of the whole abdomen, which increased up to the time when she first came under my observation, in January, 1890. She had no pain at any time, her only inconvenience arising from the size of the accumulation in the abdominal cavity, which interfered with respiration and prevented her from lying down or walking about with any comfort. Her general health was not seriously impaired, although she had lost flesh.

When seen by me her abdomen was as large as that of a woman at the full term of pregnancy. External examination showed the abdominal cavity to be filled with ascitic fluid. In the right inguinal region could be felt a hard tumor, very movable, floating in the fluid and giving to the hand a very marked sensation of *ballottement*. Its attachment was evidently on the right side, as it could not be pushed beyond the median line. The tumor could also be felt from the vagina, but its attachment could not be made out. Diagnosis, solid tumor floating in ascitic fluid, the nature of the tumor not determined.

*Operation January* 20, 1890.—Present, Drs. Kendrick and Harris and the class of students of the Atlanta Medical College. The abdomen was opened in the median line and a quantity of ascitic fluid evacuated, estimated at three gallons. The tumor, which was free from adhesions, was lifted out of the abdomen. It was attached to the right broad ligament by a slender pedicle consisting of the normal attachments of the ovary. The pedicle was transfixed and tied with silk. The abdomen was sponged out and about a pint of gelatinous material removed. No drainage. The abdominal incision was closed and dressed in the usual manner. The patient made a rapid recovery, although her temperature rose to 103.4 on the evening of the second day. She returned to her home completely cured on the 15th of February.

The tumor was oval in shape and had a smooth, white surface. On section it was found to be perfectly solid and closely resembled in appearance a fibroid tumor of the uterus. It weighed three and one-half pounds. At the point where the pedicle was divided on its lower side could be seen the remains of the ovary from whose surface the tumor appeared to grow. A microscopical examination was made by Dr. M. B. Hutchins, of Atlanta, who has kindly furnished me the following report: "I have examined sections both from frozen and from hardened portions of the specimen which you left with me. The disease is sarcoma and the tumor is made up of both small round and small spindle cells. Blood vessels are quite numerous in the round celled portions, while I could see none among the spindle cells, which were more closely packed together."

CASE XV.—BATTEY'S OPERATION. RECOVERY.—L. R., married, age 34, nullipara. For several years patient has been conscious of a "lump" in her abdomen, which of late has become painful and confined her to bed. Her menses have increased in quantity and duration until they flow nearly all the time. Examination revealed a fibroid tumor of the uterus as large as two fists.

*Operation March* 11, 1889.—Present, Drs. Harris, Lawshe and Paine. Incision in median line. All the pelvic organs completely matted together by adhesions. The right ovary was with difficulty separated and removed. The left one could not be found. Abdomen washed out and closed without drainage. Time of operation fifty minutes. Uninterrupted recovery. Highest temperature 100.6, on the evening of the second day. Patient continues to menstruate, but the flow is normal as regards quantity and duration. She is not entirely free from pain, but is much relieved, and is able to work. There has been no appreciable diminution in the size of the tumor.

CASE XVI.—SUPERFICIAL PAPILLOMA OF BOTH OVARIES. OVARIOTOMY. RECOVERY.—M. P., age 62, married, multipara.

Menopause occurred at about 45 years of age. Health has always been good. In November, 1889, she first noticed a general enlargement of her abdomen. This enlargement increased with great rapidity, so that when she came under my care, early in March, she was larger than a woman at full term of pregnancy, and was unable to stand up or lie down, but could only sit propped up in bed. She had no pain beyond the inconvenience arising from the bulk of the abdominal accumulation, and, strange to say, her general health had not suffered apparently.

External examination showed the abdomen to be filled with ascitic fluid. No tumor could be felt through the abdominal walls. By vaginal examination an obscure mass could be made out in the pelvis, on each side of the uterus. Careful investigation failed to elicit any evidence of cardiac, renal or hepatic disease. There was no œdema of any part of the body. The urine was normal. No diagnosis was made. As the symptoms pointed to some diseased condition within the abdomen, it was decided to make an exploratory incision.

*Operation March* 15, 1890.—Present, Drs. Harris, Murphey and Kennedy. The abdomen was opened in the median line and a large quantity of ascitic fluid evacuated. Upon exploring the cavity the surface of both ovaries was found to be completely covered with papillomatous growths identical in appearance with cauliflower cancer of the cervix. The left ovary contained a cyst as large as an English walnut, entirely independent of the papilloma. There was no papillomatous growth within or upon the cyst. The right ovary was as large as a goose egg, while the left was as large as a man's fist. The disease was confined entirely to the ovaries, and there were no adhesions to neighboring organs. The peritoneum was normal as far as it was visible during the operation. Both ovaries were removed, the abdomen washed out and closed without drainage. The patient made a good recovery; her highest temperature being 100.2, on the evening of the first day.

On the 25th of April there was a reaccumulation of ascitic fluid in the abdomen, to such an extent as to necessitate tapping and to render it obvious that there was recurrence of the disease within the pelvis. She returned to her home at the North about the middle of May. There is every probability that the recurring disease will rapidly prove fatal.

CASE XVII.—OVARIOTOMY. RECOVERY.—E. S., single, age 32. Had suffered for about a year with profuse hemorrhages, which had exhausted her strength to such a degree that she had become a chronic invalid. For the past few months pain in the left inguinal region had become a prominent symptom. Examination revealed a fluctuating tumor of the left ovary as large as a small orange.

*Operation March* 13, 1890.—Present, Drs. Bowne, Harris, Kennedy and C. D. Roy. Incision in the median line. The tumor, a monocyst of the left ovary, removed without difficulty. The right ovary was cirrhotic, and was also removed. Abdomen closed without washing or drainage. Time of operation, 35 minutes. Patient made a good recovery. Highest temperature 101, on the evening of the second day. Since the operation she has entirely regained her strength, and there has been no return of pain or hemorrhage.

CASE XVIII.—BATTEY'S OPERATION. RECOVERY.—G. M., age 38, married, nullipara. For three years has noticed a gradually increasing tumor in the hypogastric region, which of late has become painful and has interfered with sleep and locomotion. General health good. Examination revealed a fibroid tumor of the uterus as large as a fœtal head.

*Operation May* 24, 1890.—Present, Drs. Harris, Kennedy, Jones, and Watkins of Montgomery, Ala. The left ovary was cystic and completely bound down by adhesions which were separated with great difficulty. The right ovary was partially adherent. Both were removed. The abdomen was washed out with hot water and a drainage tube inserted. Time of operation one hour and twenty minutes. The tube was removed at the end of thirty-six hours. Patient made a good recovery. Highest temperature 100.8, on the evening of the second day.

CASE XIX.—BATTEY'S OPERATION. RECOVERY.—R. L., married, age 42, two children. For three years has complained of dragging sensation in the pelvis, with pain in walking. General health has begun to fail in the last few months. Examination revealed a fibroid tumor of the uterus as large as two fists.

*Operation May* 29, 1890.—Present, Drs. Harris, Kennedy, Elkin, and Bardwell of Talbotton, Ga. Incision in the median line. Both ovaries removed and found to be cystic. The abdomen was closed without washing or drainage. Patient made a good recovery. Highest temperature 99.6, on the evening of the second day.

CASE XX.—BATTEY'S OPERATION. RECOVERY.—L. K., single, age 22. Has always suffered from dysmenorrhœa. For the past two years has had continuous pelvic pain, which has so impaired her health as to confine her to her room. Great tenderness over the whole abdomen. Examination showed evidences of old attacks of pelvic inflammation.

*Operation June* 3, 1890.—Present, Drs. Harris, Kendrick and Elkin. Incision in the median line. Both ovaries cystic and bound down by adhesions, but easily separated and removed. Abdomen closed without washing or drainage. Patient made a good recovery. Highest temperature 100.2, on the evening of the second day. She is now free from pain and is able to walk about without discomfort.

CASE XXI.—CHOLECYSTOTOMY. RECOVERY.—The patient, a woman 28 years of age, the mother of five children, had always been well until about seven years ago, when she began to suffer with attacks of "bilious colic." In the intervals between the attacks her health was good. About five years ago she first noticed a lump in the right side below the ribs, which gradually increased in size up to the present time. Her general health began to fail about four years ago, and weakness and emaciation increased, until she was confined to the bed nearly all the time. The appetite remained fair; the bowels were constipated. There was no jaundice at any time. She had no pain, except during the paroxysms of colic.

On examination an elastic fluctuating tumor could be felt on the right side, commencing at the lower border of the liver, and extending obliquely downward and to the left as far as the umbilicus. It was apparently about five inches long, and about two inches broad. The lower extremity could be pushed upward and to the left in a circle, of which the upper extremity of the tumor was the axis. It was tender to the touch. It was apparently attached to the liver by its upper extremity. A diagnosis was made of distention of the gall bladder from obstruction, and it was decided to perform cholecystotomy, with a view to its relief.

*Operation June* 7, 1890.—Present, Drs. Rosser and Gwinn, of Conyers, and Dr. Gibson, of Newton county. An incision was made in the median line, extending two inches above the navel and and one inch below it. The distended gall bladder at once came into view. It was punctured with a trocar and half a pint of clear serum evacuated. The opening was then enlarged with scissors and a finger introduced, which came into contact with a mass of gall stones, which were removed to the number of thirty-eight. The finger was then passed down to the orifice of the cystic duct, and a stone found lying impacted in the duct with a firm constriction between it and the gall bladder. This stone could not be removed by manipulation, and therefore a bistoury was passed down to the constriction and its edges slightly nicked at various points. By further effort the stone was then dislodged and removed through the gall bladder. Examination of the cystic and common ducts showed that no other obstruction was present. The incision in the gall bladder was closed by a continued Lembert suture of cat-gut, and dropped back into the abdomen. The incision in the abdominal wall was closed and dressed in the usual manner. The patient made a good recovery without a single unfavorable symptom. Her highest temperature was 100.5, on the evening of the second day, and fell to normal on the fourth day.

CASE XXII.—OVARIOTOMY. RECOVERY.—L. K., unmarried, age 28. For four years has been gradually failing in health. For the last two years pelvic pain has been a prominent symptom. Great emaciation and weakness. Examination revealed a fluctuating tumor as large as a goose egg in the left ovarian region.

*Operation June* 10, 1890.—Present, Drs. Harris, Bowne and McRae. Incision in the median line. Cyst of left ovary as large as a goose egg. No adhesions. Right ovary normal. Both removed. The abdomen was washed out with hot water and closed without drainage. Time of operation forty minutes. In the latter part of the operation the pulse failed and patient was put to bed in a state of collapse. Stimulating injections and the external application of heat caused her to rally, and she made a good recovery. Highest temperature 100.2, on the morning following the operation.

In all these operations strict antisepsis was observed. Drainage was used in only one case and then the tube was of rubber. No opiates were given in any case. Pain and tympanites were successfully treated by Epsom salts. All sutures and ligatures were of carbolized silk except in Case XXI. The abdominal incision was closed by sutures passed through the whole thickness of the abdominal wall and removed on the tenth day. Stitch abscess and subsequent hernia occurred in one case and were probably due to a vitiated atmosphere. No food or drink was given for the first twenty-four hours; on the second day water, milk and ice, and afterwards as much nourishing food as possible. The bowels were moved on the third day with a dose of castor oil or Epsom salts. Of the twenty-two abdominal sections which I have performed two have proved fatal, the second and the fifth. My total mortality, therefore, is 9 1-11 per cent., and my last seventeen operations have been without a death.

